# Botulinum Neurotoxins Can Enter Cultured Neurons Independent of Synaptic Vesicle Recycling

**DOI:** 10.1371/journal.pone.0133737

**Published:** 2015-07-24

**Authors:** Sabine Pellett, William H. Tepp, Jacob M. Scherf, Eric A. Johnson

**Affiliations:** Department of Bacteriology, University of Wisconsin-Madison, Madison, Wisconsin, 53706, United States of America; United States Army Medical Research Institute of Infectious Diseases, UNITED STATES

## Abstract

Botulinum neurotoxins (BoNTs) are the causative agent of the severe and long-lasting disease botulism. At least seven different serotypes of BoNTs (denoted A-G) have been described. All BoNTs enter human or animal neuronal cells via receptor mediated endocytosis and cleave cytosolic SNARE proteins, resulting in a block of synaptic vesicle exocytosis, leading to the flaccid paralysis characteristic of botulism. Previous data have indicated that once a neuronal cell has been intoxicated by a BoNT, further entry of the same or other BoNTs is prevented due to disruption of synaptic vesicle recycling. However, it has also been shown that cultured neurons exposed to BoNT/A are still capable of taking up BoNT/E. In this report we show that in general BoNTs can enter cultured human or mouse neuronal cells that have previously been intoxicated with another BoNT serotype. Quantitative analysis of cell entry by assessing SNARE cleavage revealed none or only a minor difference in the efficiency of uptake of BoNTs into previously intoxicated neurons. Examination of the endocytic entry pathway by specific endocytosis inhibitors indicated that BoNTs are taken up by clathrin coated pits in both non pre-exposed and pre-exposed neurons. LDH release assays indicated that hiPSC derived neurons exposed consecutively to two different BoNT serotypes remained viable and healthy except in the case of BoNT/E or combinations of BoNT/E with BoNT/B, /D, or /F. Overall, our data indicate that previous intoxication of neuronal cells with BoNT does not inhibit further uptake of BoNTs.

## Introduction

Botulinum Neurotoxins (BoNTs) are produced by the gram positive anaerobic bacteria *Clostridium botulinum* and are the causative agent of human and animal botulism. The toxins can enter the human circulation by several routes, including ingestion through the consumption of contaminated foods, injection of the toxin, or by absorption of BoNTs produced by *C*. *botulinum* growing in an infected wound or in an infant’s intestine [1]. Once within the circulation, the toxins distribute to and efficiently enter neurons of the peripheral nervous system, in particular motor-neurons. Cell entry of BoNTs is mediated via the 100 kDa heavy chain (HC), which is linked via a disulfide bond to the 50 kDa LC. The LC enters the cell’s cytosol, where it cleaves a soluble N-ethylmaleimide-sensitive factor attachment protein receptor (SNARE) protein, thereby preventing formation of a functional SNARE complex and fusion of the synaptic vesicles with the pre-synaptic cell membrane [2]. This results in an inability of the cell to release neurotransmitter, thereby causing the flaccid paralysis characteristic of botulism. The enzymatically active LC remains inside the cytosol for a prolonged period of time and continues to cleave newly synthesized SNARE proteins [3].

BoNTs have been categorized into seven immunologically distinct serotypes (A-G) [4], and an eighth serotype has recently been proposed (H) [5, 6]. In addition to being immunologically distinct, the serotypes have several unique characteristics, including distinct SNARE target sites, specific cell surface receptors, and distinct durations of action [3]. BoNT/A, E, and C all cleave SNAP-25 (synaptosomal-associated protein of 25 kDa) at distinct sites, whereas BoNT/B, D, F, and G and the putative H cleave VAMP 1 and 2 (vesicle-associated membrane protein (also known as synaptobrevin) at distinct sites [7]. BoNT/C also cleaves syntaxin [7]. The specific neuronal cell entry of BoNTs is mediated by binding of the toxins to gangliosides and protein receptors [8]. All BoNT serotypes bind to specific polysialo-gangliosides, which are enriched in the outer leaflet of the neuronal cell membrane, and this association is essential for cell entry of the toxins [8]. In addition, several of the BoNT serotypes have been found to bind to the synaptic vesicle proteins SV2 (BoNT/A, /E, and possibly /D) or synaptotagmin I and II (BoNT/B, G, /DC), and this association appears essential for cell entry [8]. It is of particular interest that the BoNT binding site of synaptic vesicle proteins has been identified to be located on a luminal domain of these proteins, which is located on the inside of synaptic vesicles [8].

Due to the requirement of the toxins to bind to synaptic vesicle proteins, combined with the observation that chemical stimulation of neurons increases neuronal uptake of several BoNT serotypes [9–14], it has been hypothesized that cell entry of BoNTs is dependent on active synaptic vesicle recycling. Synaptic vesicle exocytosis results in display of the intravesicular domains of synaptic vesicle proteins on the cell membrane, thus allowing binding of BoNTs. In fact, pre-exposure of primary rat hippocampal neurons to BoNT/B to block synaptic vesicle exocytosis, followed by exposure to BoNT/A, has been reported to eliminate BoNT/A cell binding [15]. In addition, pre-treatment of primary rat cortical neurons with BoNT/D to block synaptic vesicle recycling has been shown to significantly decrease depolarization-dependent endocytic uptake of the BoNT/A heavy chain receptor binding region (HCR) [16]. On the other hand, primary mouse spinal cord (MSC) neurons or rat cortical neurons that have been previously intoxicated with BoNT/A and in which about 50% of the SNAP-25 was cleaved, have been shown to uptake BoNT/E, which was demonstrated by assessing the different SNAP-25 cleavage products [17, 18]. While it could be argued that 50% of SNAP-25 cleavage only partly blocked exocytosis of synaptic vesicles, thereby still allowing some synaptic vesicle endocytosis for uptake of BoNT/E, another independent study dissociated synaptic vesicle endocytosis from exocytosis in BoNT/A intoxicated neurons [19]. In this elegant study, primary mouse spinal cord cells pre-exposed to BoNT/A, and in which a complete absence of exocytosis was demonstrated, were still able to take up FM1-43 dye or HRP by synaptic vesicle endocytosis. Pre-exposure of the cells to tetanus toxin or BoNT serotypes B, C, and D, on the other hand, did not result in dye or HRP uptake. This led the authors to conclude that BoNT/A is unique in that it is able to un-couple synaptic vesicle exocytosis and endocytosis [19]. However, five cycles of chemical depolarization of cells pre-exposed to BoNT/A prevented following dye uptake, indicating that the membrane available for endocytosis is limited in these cells [19].

Sequential cell entry by BoNT/A and /E has also been explored *in vivo*, making use of the much faster recovery after BoNT/E injection than after BoNT/A injection. Experiments in rats sequentially injected with BoNT/E and BoNT/A or BoNT/A and BoNT/E resulted in slow recovery similar to BoNT/A alone as determined by twitch tension assay [18]. Neuronal cell entry of BoNT/A followed by entry of BoNT/E was further demonstrated by sequentially exposing hemidiaphragm muscle preparations and selectively blocking BoNT/A with 3,4-DAP. This resulted in a recovery similar to BoNT/A and not BoNT/E, confirming sequential cell entry of the toxins [18]. Similarly, mice injected into the gastrocnemius muscle with combinations of BoNT/A and either BoNT/B or BoNT/E recovered more slowly than mice injected with only BoNT/A as determined by a voluntary wheel running assay [20]. In contrast, a small study examining recovery from inhibition of compound muscular action potentials after local injection of combinations of BoNT/A and BoNT/E into the extensor digitalis brevis muscle of human volunteers indicated recovery similar to that of BoNT/E [21]. While these data disagree with the previous two studies on duration of action, they still indicate uptake of BoNT/E into neurons that also have taken up BoNT/A.

BoNTs that have been catalytically inactivated have been suggested as specific neuronal drug delivery vehicles [22–25]. Such molecules hypothetically would present ideal drug delivery vehicles to deliver potential BoNT LC inhibitors into neurons intoxicated by BoNT. However, the cell-based and *in vivo* data on potential BoNT re-entry into BoNT intoxicated neurons are conflicting as described above. In addition, the disruption of synaptic vesicle recycling by BoNTs and thus the presumed inability of BoNTs to enter neurons already intoxicated by BoNT has been considered to compromise such approaches. Here we examined the entry of BoNTs into neurons already intoxicated by several other BoNT serotypes (A, B, D, E, and F) in a quantitative cell-based assay using SNARE cleavage as an endpoint. The data show that BoNTs enter human neurons previously intoxicated with other BoNT serotypes with similar or only slightly reduced efficiency during both continuous and activity-dependent cell entry.

## Materials and Methods

### Ethics Statement

All work described in this manuscript was approved by the University of Wisconsin-Madison Institutional Biosafety Committee. All animal experiments were approved by and conducted according to guidelines by the University of Wisconsin Animal Care and Use Committee. The accepted mouse bioassay was employed to determine the specific activity of botulinum neurotoxins used in this study. This assay is the only procedure approved by the FDA to detect the presence of botulinum toxins, and no alternative in vitro method has been accepted by the FDA for detection of BoNTs. This assay using death as the final endpoint has been specifically reviewed and approved by the University of Wisconsin-Madison Institutional Biosafety Committee. Cell-based or *in vitro* assays are used wherever possible in our laboratory and were used for all experiments in this study except for specific activity determination.

### Botulinum Neurotoxin and Reagents

The 150 kDa BoNT/A1, /B1, /D1, /E1, and /F1 were isolated from *C*. *botulinum* strains Hall A-*hyper*, Okra B, D1873, Beluga E, and Langeland F as previously described [26, 27]. The specific activity of the toxins was determined by the standard intraperitoneal (IP) mouse bioassay as previously described [28, 29]. Specific activity in mice was 1.25 x 10^8^ mouse IP LD50 Units (U) / mg (A1), 5 x 10^8^ U/mg (B1), 1.1 x 10^8^ U/mg (D1), 8.2 x 10^7^ U/mg (E1), and 3.3 x 10^7^ U/mg (F1). For the mouse bioassay, groups of 4 mice were injected intraperitoneally with 6 serial dilutions of each toxin, and monitored for symptoms for up to 4 days. The endpoint of the assay involves typical botulism symptoms with death of the mouse to indicate the presence of a certain amount of toxin (mouse lethal dose). Since mice that show symptoms can recover, it is usually not feasible to terminate the assay. Typically, approximately 50% of the mice used in the assay survive. No drugs are administered because the goal of the experiment is to determine the specific activity (LD50) of the purified toxin. Mice are observed at least twice daily, including within 2 to 4 h of injection, prior to leaving for the day, and immediately upon arrival in the morning. Mice exhibiting symptoms are monitored more frequently in order to accurately evaluate the presence of botulinum toxin. Dead mice are removed from cages immediately upon observation, and very sick mice that are expected to die as well as any surviving mice at the end of the study are humanely euthanized by carbon dioxide asphyxiation in accordance with OLAW guidelines.

The endocytosis inhibitors nystatin, chlorpromazine hydrochloride, dansylcadaverine, cholesterol oxidase, 5-(N,N-dimethyl)-amiloride hydrochloride (DMA), and cytochalasin D were all purchased from SIGMA. Stock solutions were prepared in DMSO at the following concentrations and stored at -20°C: Nystatin: 5.4 mM, chlorpromazine: 10 mM, dansylcadaverine: 30 mM, cholesterol oxidase: 1000 U/ml, DMA: 20.4 mM, and cytochalasin D: 5 mg / ml. All tissue culture reagents, SDS-PAGE, and Western blot supplies were purchased from Life Technologies unless otherwise noted.

### Neuronal Cell Models

The iCell Neurons and media were purchased from Cellular Dynamics Inc. (Madison, WI) and seeded and maintained according to company instructions. Primary mouse (strain ICR, at E12) spinal cord cells were prepared as described previously and were seeded and maintained in serum free culture medium (Neurobasal medium supplemented with 2% B27, 2 mM glutamax, and 100 units/mL penicillin/streptomycin (all from Invitrogen)) [30, 31]. Both cell types were seeded at about 40,000 cells per well into 0.01% poly-L-ornithine (SIGMA) and 8.3 μg/cm^2^ matrigel (BD Biosciences) coated 96-well Techno Plastic Products (TPP) plates. The primary cells were allowed to mature for 18 days before use, and iCell Neurons were used at day 5 in culture.

### Cell-based Assays

The cells were exposed to the following concentrations of BoNT in 50 μl of the respective media (unless noted otherwise) for 48 h to achieve complete or nearly complete SNARE cleavage (10 U (mouse IP LD50 Units)/ well BoNT/A, 1000 U / well; BoNT/B, 1,000 U / well; BoNT/D, 2,000 U / well; BoNT/E, and 30 U / well BoNT/F). For consecutive exposures, the extracellular solution containing BoNT was removed after 48 h, and the cells were carefully washed twice in 300 μl culture medium. The cells were then exposed to serial dilutions of the indicated BoNT for 48 h in 50 μl of the respective culture medium. In parallel, cells not pre-exposed to BoNT were exposed to the same serial dilutions as indicated. All samples were tested in triplicate. After 48 h, toxin was removed and cells were lysed in 1X lithium dodecyl sulfate (LDS) sample buffer (Invitrogen). For the consecutive exposure of neurons to BoNT/E followed by BoNT/A, cells were incubated for a further 3 weeks after the removal of BoNT/A dilutions before harvest, to allow for recovery of BoNT/E-cleaved SNAP-25. Cell lysates were analyzed by Western immunoblot for SNAP-25 and VAMP2 cleavage as previously described using a monoclonal anti-VAMP2, syntaxin, or anti-SNAP-25 antibody from Synaptic Systems (Göttingen, Germany) [12, 31]. The anti-SNAP-25 antibody recognizes both cleaved and uncleaved SNAP-25 equally, and the VAMP2 antibody detects only uncleaved VAMP2, which is quantitated in relation to syntaxin. SNAP-25, VAMP2 and syntaxin bands were quantified by densitometry using a Foto/Analyst FX system and TotalLab Quant software (Fotodyne). Data plots and EC50 values (four parameters–variable slope) were generated using PRISM 6 software, and statistical significance was determined by PRISM 6 software using an Extra-sum-of-squares F-test with an α-value of 0.05 and comparing the logEC50 values and Hill slopes. Two curves were deemed significantly different if the p value was below 0.01.

For analysis of depolarization dependent uptake, the previously BoNT-exposed neurons were washed, and these as well as not previously exposed neurons were exposed to serial dilutions of BoNT in cell stimulation media (modified neurobasal medium formulated to contain 56 mM KCl and 2.2 mM CaCl_2_ and supplemented with B27 and glutamax, all from Invitrogen) for 5 min. at 37°C, followed by removal of the toxin and washing the cells twice with 300 μl culture media. The cells were then incubated for another 24 h in culture media to allow for SNAP-25 or VAMP2 cleavage. Cells were harvested in 50 μl LDS sample buffer and lysates analyzed as above.

To analyze the effect of endocytosis inhibitors on BoNT uptake, iCell Neurons were first exposed to 1,000 U of BoNT/B for 48 h, followed by toxin removal and washing of the cells as above. The neurons were then exposed to the endocytosis inhibitors at 37°C for 30 min at the following concentrations, which have previously been shown to inhibit endocytosis [32–36]: nystatin: 54 μM, chlorpromazine: 100 μM, dansylcadaverine: 300 μM, cholesterol oxidase: 10 U/ml, DMA: 204 μM, and cytochalasin D: 50 μg / ml. The positive and negative control cells were exposed to culture media containing 1% DMSO. After 30 min., the inhibitors were removed and cells exposed to 200 U/well of BoNT/A1 in either cell stimulation media or in culture media for 7.5 min and 10 min, respectively, at 37°C. Toxin was removed, cells washed twice with 300 μl of culture media, and incubated for another 7 h to allow for SNAP-25 cleavage. Cells were harvested in 50 μl LDS sample buffer and lysates analyzed as above.

### Cytotoxicity Assay

In order to assess the possible cytotoxic impact of intoxication of iCell Neurons with two BoNT serotypes that resulted in cleavage of both SNAP-25 and VAMP2, cells were first exposed to 5 nM of BoNT/A or BoNT/E and allowed to uptake toxin for 24 h at 37°C, 5% CO_2_. After 24 h, cells were washed twice with 300 μl culture media and exposed to 5 nM of BoNT/B, /D, or /F and incubated at 37°C, 5% CO_2_ for an additional 24 h. All were tested in triplicate. One set of cells was lysed in 50 ul of 1x lithium dodecyl sulfate (LDS) sample buffer (Invitrogen) and analyzed for SNAP-25 and VAMP2 cleavage via Western blot as previously described [30, 31]. To examine cell lysis, cells were visually observed for distress by light microscopy, and LDH release into the culture media was monitored daily for the next 3 days by using the lactate dehydrogenase (LDH)-dependant Cytotox 96 Non-Radioactive Cytotoxicity Assay according to the manufacturer's instructions (Promega, Fitchburg, WI). For the 100% lysis control, lysis solution was added to the culture media 24 h before the assay on each day, such that the LDH release was determined during the time periods of 0–24 h, 24–48 h, and 48–72 h after the addition of the second BoNT serotype. Each sample point was measured in triplicate, and a student t-test was used to analyze statistical significance of LDH release as compared to the background control (no toxin added to cells).

## Results

### Pre-exposure to BoNT did not result in significant changes in sensitivity to subsequent BoNT intoxication in cultured neurons

**Fig 1:**
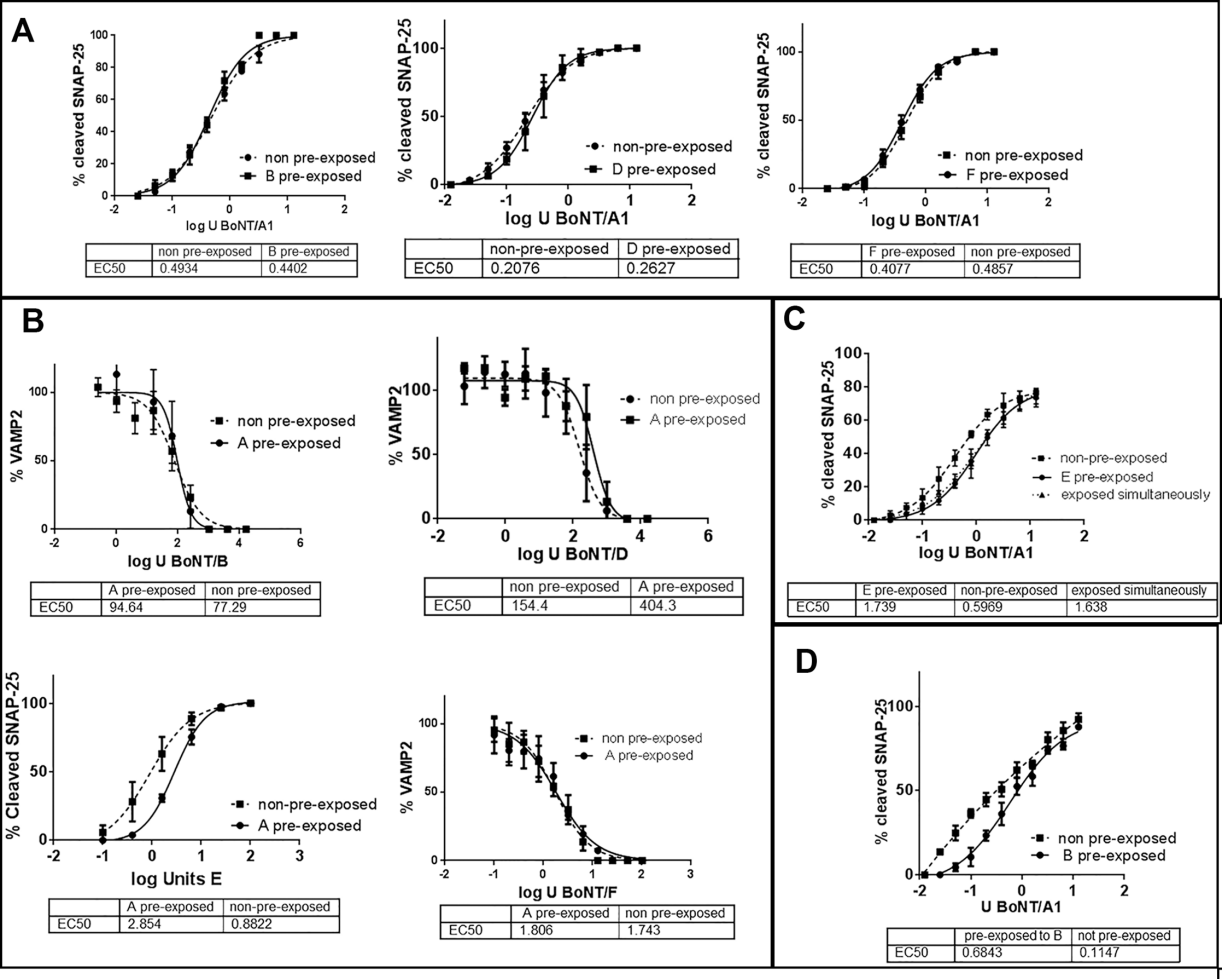
Sequential uptake of BoNTs. Human iPSC derived neurons (iCell Neurons) (a, b, c) or primary mouse spinal cord cells (d) were exposed to the indicated BoNT serotypes (10 U / well BoNT/A, 1000 U / well BoNT/B, 1,000 U / well BoNT/D, 2,000 U / well BoNT/E, and 30 U / well BoNT/F) for 48 h to cause full SNARE cleavage (see S1 Fig). After complete removal of all extracellular toxin, the pre-exposed and not pre-exposed neurons were exposed to serial dilutions of the indicated BoNTs for 48 h in parallel, and cell lysates were analyzed for SNARE cleavage by Western blot using an anti-SNAP-25 antibody that recognizes both cleaved and uncleaved SNAP-25 equally and a VAMP2 antibody that detects only uncleaved VAMP2, which is quantitated in relation to syntaxin (all antibodies from Synaptic Systems). The graphs show the quantitative analyses of SNARE cleavage by the second BoNT serotype (as shown on the x-axis label), and the graph legends indicate the BoNT serotype added for pre-exposure. C: Cells first exposed to BoNT/E followed by exposure to BoNT/A were incubated for an additional 3 weeks before cell lysis to allow for recovery of BoNT/E cleaved SNAP-25. In all cases, each data point was measured in triplicate, and data were analyzed for statistical significance using an F-test in PRISM 6 software (n = 3).

In order to determine the impact of BoNT pre-exposure on subsequent BoNT intoxication, human induced pluripotent stem cell (hiPSC) derived neuronal cells were exposed sequentially to two different BoNT serotypes. The first BoNT serotype was added at concentrations previously determined to result in 100% or nearly 100% of SNARE cleavage [12], and after complete removal of the toxin and washing of the cells, serial dilutions of the second BoNT were added to the pre-exposed cells as well as to non-exposed cells. Cell populations were assessed for SNAP-25 or VAMP2 cleavage via Western blot and densitometry to confirm 100% SNARE cleavage by the first BoNT serotype and determine the EC50 values (the value at which 50% of the maximum effect was achieved) of the second BoNT serotype. Pre-exposure to BoNT/B, /D, or /F did not significantly alter the EC50 value for SNAP-25 cleavage by BoNT/A in the pre-exposed versus non pre-exposed cells (Fig 1A). Similarly, pre-exposure to BoNT/A at concentrations that resulted in full SNAP-25 cleavage did not significantly alter the EC50 values for VAMP-2 cleavage caused by subsequent exposure to serial dilutions of BoNT/B, /D, or /F (Fig 1B). A slight decrease (about 3-fold) in the EC50 value of BoNT/E induced SNAP-25 cleavage was observed in cells that had been pre-exposed to BoNT/A (Fig 1B).

Because BoNT/E cleaves a larger amino acid fragment from the C-terminus of SNAP-25 than BoNT/A, and BoNT/E cleaved SNAP-25 recovers within 2–3 weeks, a modification of the above assay was used to evaluate BoNT/A entry into hiPSC derived neurons previously intoxicated by BoNT/E. In this assay, the cells were first exposed to BoNT/E followed by exposure to serial dilutions of BoNT/A. However, instead of harvesting the cells after 48 h, only one set of cells was harvested to confirm SNAP-25 cleavage by BoNT/E, respectively, and for the other triplicate sets the toxin was removed and cells were incubated in toxin-free medium for another 3 weeks before harvesting to allow for recovery of BoNT/E cleaved SNAP-25. In addition, simultaneous exposure of BoNT/E and BoNT/A was also assessed by exposing the cells to 2,000 U (mouse IP LD50 Units)/ well of BoNT/E (enough to cause 100% SNAP-25 cleavage) combined with serial dilutions of BoNT/A. The EC50 values for both, BoNT/E pre-exposed and simultaneously exposed cells were about 3-fold lower than those of not pre-exposed cells (Fig 1C).

Since the above data were derived from hiPSC derived neuronal cultures for which characteristics such as synaptic activity and neurotransmitter release have not been studied in detail, primary mouse spinal cord cells (MSC cells) were employed in a similar assay as a second cell model. MSC cells were pre-exposed to BoNT/B before intoxication with serial dilutions of BoNT/A. No difference in the EC50 value for BoNT/A activity was observed in BoNT/B pre-exposed and not pre-exposed cells (Fig 1D), indicating that the above observations are not restricted to only one cell model.

Even though toxin concentrations sufficient to result in about 100% SNARE cleavage were chosen for the BoNT pre-exposure, the effect of even greater amounts of toxin were assessed by pre-exposing hiPSC derived neurons to 30 nm of BoNT/B followed by exposure to serial dilutions of BoNT/A. No significant impact on BoNT/A intoxication was observed (data not shown), indicating that the entry of BoNT/A into cells previously intoxicated with BoNT/B is independent of the concentration used for BoNT/B pre-exposure.

### Depolarization dependent BoNT uptake into cultured neurons is not significantly affected by BoNT pre-exposure of the neurons

**Fig 2:**
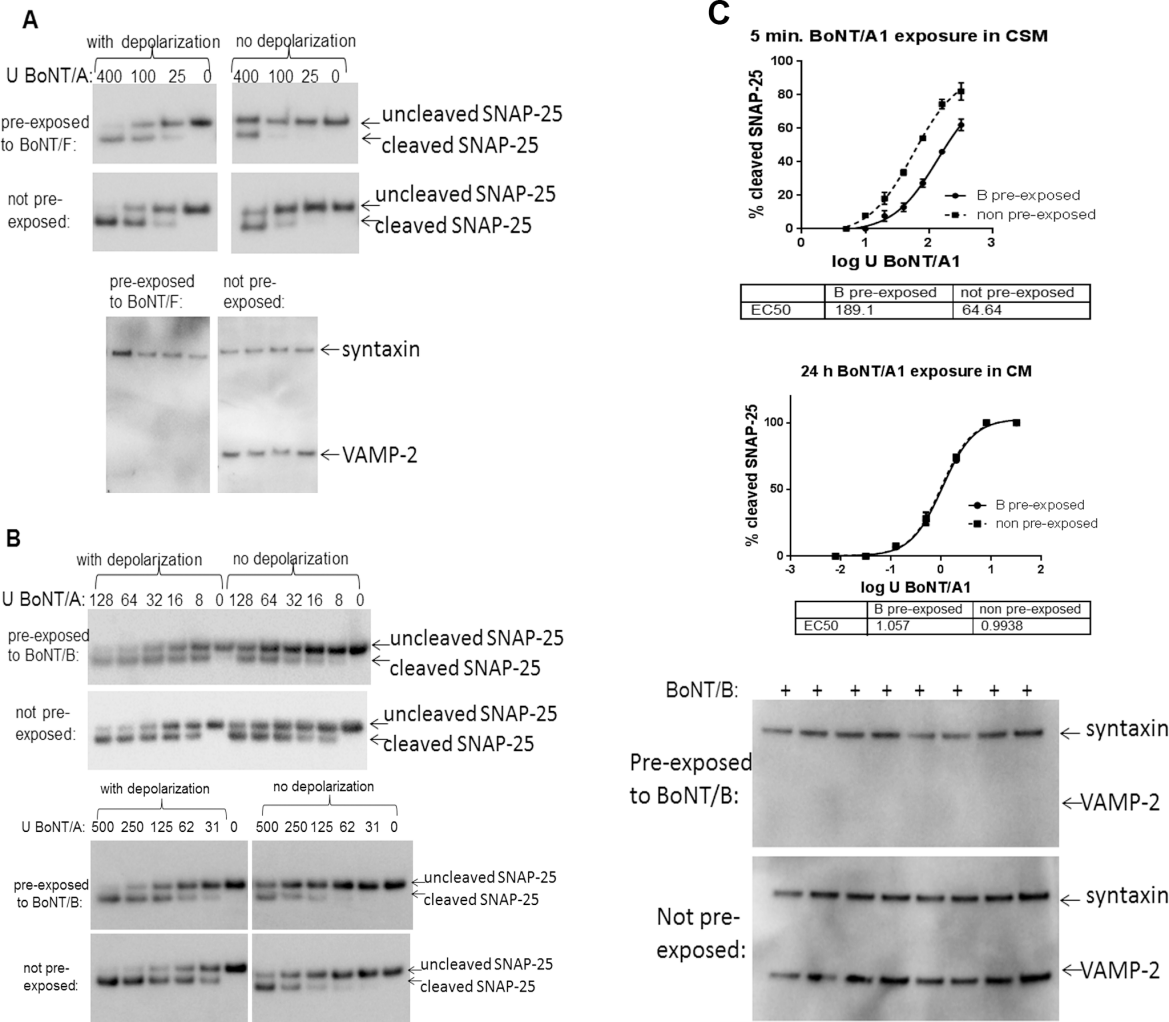
Activity dependent sequential BoNT entry into neurons. Neuronal cultures were pre-exposed to BoNT/B or BoNT/F for 48 h, followed by complete removal of extracellular toxin. The pre-exposed or not pre-exposed cells were then exposed to the indicated concentrations (U / well) of BoNT/A1 in culture media or cell stimulation media (containing 56 mM KCl and 2.2. mM CaCl2) for 5 min, followed by removal of toxin, washing of the cells, and incubation for 24 h to allow for SNAP-25 cleavage. Cell lysates were analyzed for SNARE cleavage by Western blot and densitometry. All samples were tested in triplicate. (a) Human iPSC derived neurons (iCell Neurons) pre-exposed to 30 U BoNT/F. A Representative Western blot of SNAP-25 cleavage in depolarized (cell stimulation media) or not depolarized (culture media) cells is shown in the top figure, and VAMP-2 cleavage in BoNT/F pre-treated neurons in shown in the bottom figure. (b) Primary mouse spinal cord cells (top) or iCell Neurons (bottom) were exposed to 20,000 U BoNT/B / well (MSC cells) or 1000 U BoNT/B / well (iCell Neurons) for 48 h to cleave all VAMP2, followed by exposure to the indicated concentrations (U / well) of BoNT/A1 in culture media or cell stimulation media (containing 56 mM KCl and 2.2. mM CaCl2) for 5 min. Toxin was removed, cells washed and incubation for 24 hgig. to allow for SNAP-25 cleavage. Representative Western blots are shown. (c) Human iPSC derived neurons (iCell Neurons) pre-exposed to 1000 U BoNT/B for 48 h (bottom image), and after toxin removal cells were depolarized with 5 sequentially rounds of depolarization in cell stimulation media before exposure to serial dilutions of BoNT/A1 in either cells stimulation media for 5 min, or in culture media for 24 h. The graphs depict quantitative data from triplicate samples, respectively, and statistical significance was evaluated using an F-test in PRISM6.

To assess depolarization-dependent uptake of BoNT into BoNT pre-exposed and not pre-exposed neurons, iCell Neurons were first treated with 30 U / well of BoNT/F to cleave all detectable VAMP2, followed by complete toxin removal. The pre-exposed or not pre-exposed cells were then exposed for 5 min to serial dilutions of BoNT/A in culture media or in cell stimulation media (Neurobasal media modified to contain 56 mM KCl and 2.2 mM CaCl_2,_ supplemented with B27 and glutamax), toxin was removed, cells washed, and incubated for an additional 24 h to allow for SNAP-25 cleavage. While under all of these conditions SNAP-25 cleavage occurred in a dose dependent fashion, it was significantly more efficient with depolarization as previously reported (12). There was a slight reduction (estimated about 2-fold) in depolarization dependent and non-depolarization dependent fast uptake of BoNT/A in pre-exposed neurons as compared to non pre-exposed neurons (Fig 2A). This was further investigated in both iCell Neurons and MSC cells by exposing the cells to 250 U / well (iCell Neurons) or 20,000 U / well (MSC cells) of BoNT/B resulting in complete (iCell Neurons) or nearly complete (MSC cells) VAMP2 cleavage (not shown), followed by exposure to serial dilutions of BoNT/A for 5 min with or without depolarization as described above. In both cases there appeared to be a slight reduction in SNAP-25 cleavage in pre-exposed versus non pre-exposed neurons, indicating slightly decreased fast uptake into BoNT pre-exposed neurons (Fig 2B).

A previous report indicated that endocytosis is uncoupled from exocytosis in BoNT/A treated primary mouse spinal cord cells, and after repeated depolarization of these intoxicated neurons endocytosis of the fluorescent dye FM41-3 ceases, leading to the conclusion that the membrane available for endocytosis is limited in BoNT/A intoxicated neurons [19]. To investigate this further, iCell Neurons were first exposed to 1,000 U / well of BoNT/B, resulting in complete VAMP2 cleavage (Fig 2C). After complete removal of all extracellular toxin, the pre-exposed as well as not pre-exposed cells were induced to undergo 5 rounds of depolarization by alternating 5 min incubations in cell stimulation media and culture media five times. Immediately after that, the cells were exposed to serial dilutions of BoNT/A for 5 min in cell stimulation media, followed by complete toxin removal and incubation for 24 h to allow for SNAP-25 cleavage. In addition, a second set of the same cells was exposed to serial dilutions of BoNT/A in culture media for 24 h. SNAP-25 cleavage was significantly reduced by about 3–4 fold in the pre-exposed cells after short (5 min) depolarization dependent exposure only, whereas there was no difference after the 24 h exposure (Fig 2C). This indicates slightly reduced depolarization dependent fast entry into BoNT pre-exposed neurons after multiple rounds of depolarization, but similar entry during continuous 24 h exposure.

### BoNTs enter BoNT pre-exposed neurons via clathrin-mediated endocytosis

**Fig 3:**
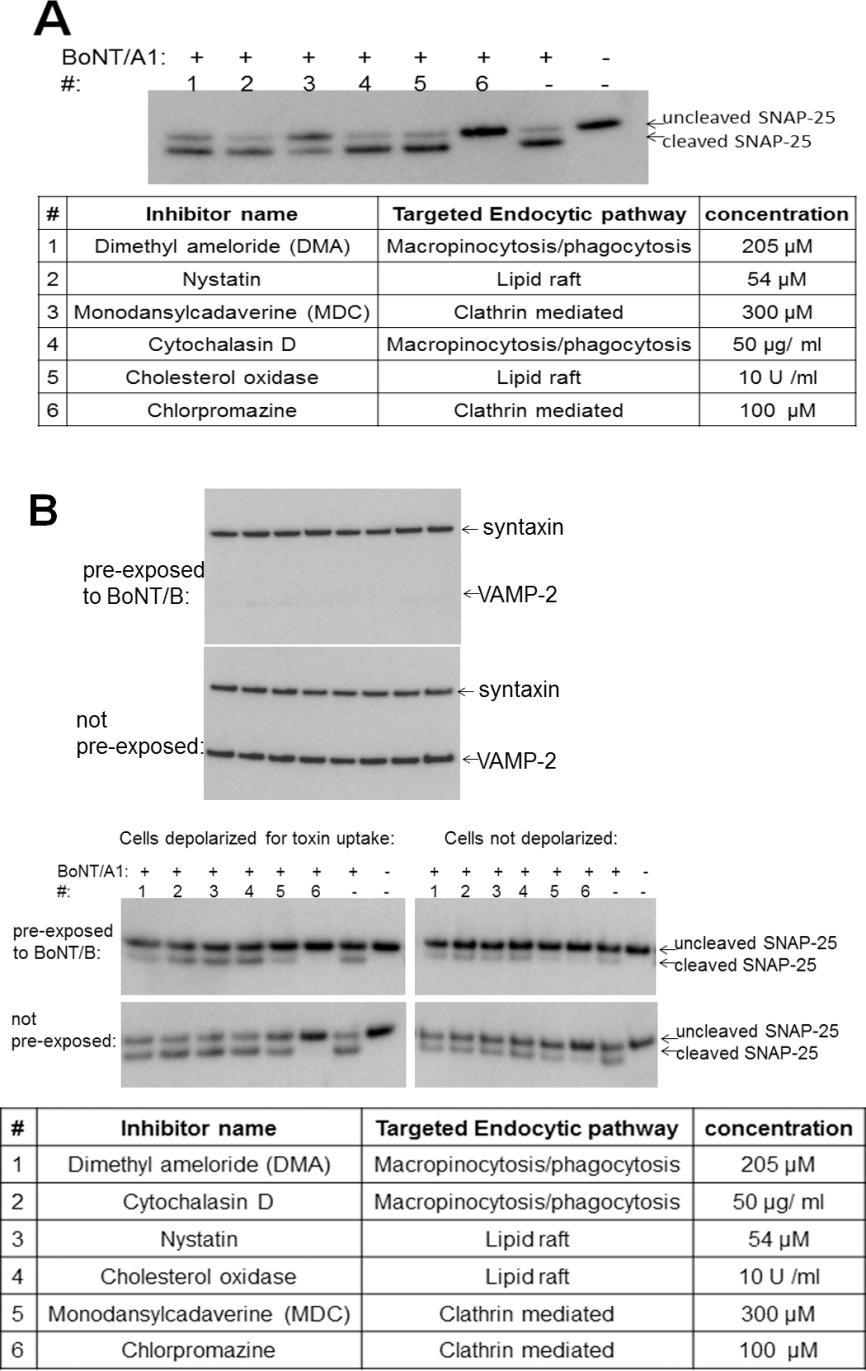
Inhibition of BoNT/A entry into human neurons by endocytosis inhibitors specific for clathrin dependent endocytosis. (a) Human iPSC derived neurons (iCell Neurons) were treated with the indicated endocytosis inhibitors for 30 min at 37°C. The cells were then exposed to 200 U of BoNT/A1 / well for 7 h, and cell lysates analyzed for SNAP-25 cleavage by Western blot. B: Human iPSC derived neurons (iCell Neurons) were exposed to 1000 U / well of BoNT/B to achieve full VAMP2 cleavage (top panel). Toxin was removed, and cells were treated with the indicated endocytosis inhibitors for 30 min at 37°C. The cells were then exposed to 200 U of BoNT/A1 / well for 5 min in either culture media or cell stimulation media, followed by toxin removal and further incubation for 8 h. Cell lysates were analyzed for SNAP-25 cleavage by Western blot. SNAP-25 cleavage in depolarized (cell stimulation media) or not depolarized (culture media) cells is shown in the Western blot, and the respective inhibitors are indicated in the table.

In order to investigate the endocytosis pathway utilized by BoNT, iCell Neurons were treated with six different specific endocytosis inhibitors prior to a 7 h exposure to BoNT/A. Two of the inhibitors, dimethylameloride (DMA) cytochalasin D, are specific inhibitors of micropinocytosis/phagocytosis, whereas nystatin and cholersterol oxidase inhibit lipid raft, including caveolar, endocytosis, and chlorpromazine and monodansylcadaverine (MDC) are inhibitors of clathrin mediated endocytosis. Analysis of SNAP-25 cleavage in the cells revealed that chlorpromazine completely inhibited SNAP-25 cleavage, and monodansylcadaverine provided partial inhibition (Fig 3A), which is consistent with earlier reports indicating that BoNTs enter cells via clathrin mediated endocytosis [19, 37–39].

To investigate whether BoNT pre-exposure of neurons alters the uptake mechanism of subsequently added BoNTs, BoNT/B pre-exposed or non-exposed neurons were treated with the different endocytosis inhibitors followed by exposure to 200 U/well of BoNT/A for 5 min with or without depolarization. After complete removal of extracellular toxin and incubation for 7 h to allow for SNAP-25 cleavage, the SNAP-25 cleavage pattern was analyzed. In both BoNT/B pre-exposed and not pre-exposed neurons, and with or without depolarization, SNAP-25 cleavage was only significantly inhibited by the inhibitors of clathrin dependent endocytosis, chlorpromazine and MDC (Fig 3B). This indicates that BoNT/A entry into both BoNT/B pre-exposed or non pre-exposed neurons proceeds via clathrin dependent endocytosis.

### Sequential exposure of cultured human neurons to BoNT/E and BoNT/B, /D, and /F results in slight cytotoxicity

**Fig 4:**
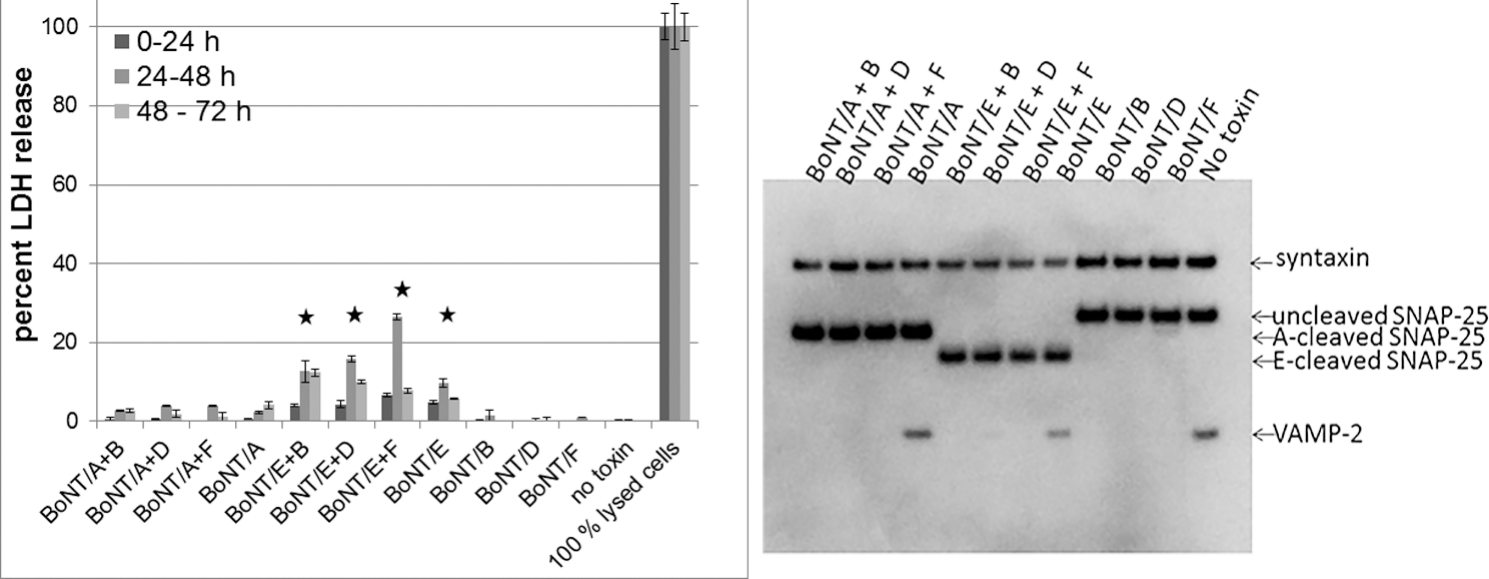
Cytotoxicity of sequential exposure of human neurons to different BoNT serotypes. Human iPSC derived neurons (iCell Neurons) were exposed to 5 nM BoNT/A or BoNT/E for 24 h, followed by sequential exposure to 5 nM BoNT/B, /D, or /F for 24 h. Culture media was examined at 0–24 h, 24–48 h, and 48–72 h post addition of the second toxin for LDH release indicating cytotoxicity. Lysis buffer for the 100% lysed cells was added at 0 h (for the 0–24 h time point), 24 h (for the 24–48 h time point), and 48 h (for the 48–72 h time point). Media was replaced between each time point. All samples were measured in triplicate, and the average and standard deviation are shown in the graph. Statistical significance in relation to the control (cells with no toxin added) was determined by students t-test. Values that were significantly different at all three time points are indicated by a * (p < 0.01). One set of cells was lysed at 24 h post addition of the second toxin, and cell lysates were analyzed for SNARE cleavage by Western blot. One representative Western blot of triplicate samples is shown.

Since we determined that it is possible for multiple BoNTs to enter the same neurons sequentially, the impact of sequential intoxication by different BoNTs on cell survival was assessed. Cells were sequentially exposed to 5 nM of BoNT/A or /E for 24 h, followed by exposure to 5 nM of BoNT/B, /D, or /F, respectively, for 24 h. SNARE cleavage was confirmed by Western blot (Fig 4A), and potential cell lysis was assessed by a lactate dehydrogenase (LDH) release assay during 0–24 h,24–48 h, and 48–72 h post addition of the second toxin (Fig 4B). Only minimal and not statistically significant LDH release (below 3%) was detected and the cells appeared healthy by microscopy when exposed to combinations of BoNT/A and /B, /D, or /F. However, exposure to 5 nM BoNT/E alone resulted in a significant amount of LDH release (about 5–9%) and mild cytotoxicity was observed by microscopic evaluation. The LDH release was strongest at 24–48 h post exposure to the second toxin, and decreased at 48–72 h (Fig 4B). Sequential exposure of BoNT/E exposed cells to BoNT/B, /D, or /F increased LDH release to 13%, 16%, and 27%, respectively at the 24–48 h interval (Fig 4B), which correlated with microscopic observations. As with BoNT/E exposure alone, the LDH release decreased after the 48 h time point. The fraction of cells that did survive appeared normal by microscopy after one week of further incubation. Interestingly, exposure to lower amounts of BoNT/E (1 nM) resulted in minimal cell death, however, sequential exposure to BoNT/B, /D, and especially /F did result in significant cell death ranging from about 10–30%, with the greatest amount of LDH release observed for the combination of BoNT/E and /F (not shown). These data indicate that at very high concentrations BoNT/E caused cell death, and that this is increased by sequential exposure to BoNT/B, /D, and especially /F. Very high concentrations of BoNT/A or combinations of BoNT/A with BoNT/B, /D, or /F, on the other hand, did not result in significant cell death.

## Discussion

The mechanism of neuronal cell entry by BoNTs is not fully understood. It has been shown that BoNTs bind to gangliosides and in most cases also to the luminal domain of synaptic vesicle protein receptors to associate with the neuronal cell membrane [8]. This leads to endocytosis, which has been shown to be via a clathrin mediated mechanism [19, 37–39], as is typical for receptor mediated cell entry. Since BoNTs cleave intracellular SNARE proteins, which are essential for synaptic vesicle exocytosis, BoNT cell entry leads to a block in synaptic vesicle exocytosis and failure to release neurotransmitter. A tight coupling of synaptic vesicle exocytosis and endocytosis has been proposed [40–42], which would predict that a block in SV exocytosis results in a block in endocytosis and thus no further uptake of BoNT. Since uptake of most BoNTs involves binding to the luminal domain of synaptic vesicle proteins [8, 10, 15, 43–46], which would be displayed on the extracellular membrane surface after synaptic vesicle exocytosis, a block in exocytosis would predict that the protein receptors for BoNTs are not displayed on the cell membrane anymore, resulting in a cessation of further BoNT binding and uptake.

In 2006, Dong et al observed that pre-exposure of cultured rat hippocampal neurons to 30 nM BoNT/B, which resulted in no detectable VAMP2 in the cells, resulted in the lack of BoNT/A cell association in mouse hippocampal and cultured spinal cord neurons [15]. However, the data presented here show no or only a minor change in BoNT/A-induced cleavage of SNAP-25 after pre-exposure to BoNT/B, /D, /E, or /F at concentrations that result in full SNARE cleavage and thus would result in a block in synaptic vesicle exocytosis (Fig 1). Even when cells were exposed to 30 nm BoNT/B, much more than required to achieve full VAMP2 cleavage, there was no observed impact on subsequent BoNT/A uptake (data not shown). The only BoNT serotype that had a minor impact on a consecutive intoxication by BoNT/A was a pre-exposure to BoNT/E. Similarly, pre-exposure of the neurons to BoNT/A did not significantly alter uptake of BoNT/B, /D, or /F, and had only a minimal effect on the uptake of BoNT/E. These data suggest that different BoNT serotypes can enter neuronal cells already intoxicated by another BoNT serotype with the same efficiency as not pre-exposed neurons. These data are in agreement with a previous publication that showed that primary mouse spinal cord cells exposed to BoNT/A were able to uptake BoNT/E [9], and expands these data to BoNT serotypes A, B, D, E, and F. BoNT serotype C could not be used in this study because it caused cytotoxicity at the required concentrations, such that consecutive exposure with another BoNT was impossible. BoNT/G was not available.

Since this re-uptake assay utilized a 48 h BoNT exposure, the requirement for synaptic vesicle recycling may have been bypassed in this experiment. Therefore, activity-dependent uptake of BoNT/A into neurons previously intoxicated with BoNT/B or /F was examined. For this assay, neurons were first exposed to BoNT/B or /F at concentrations that resulted in full SNARE cleavage, followed by 5 min. exposure to BoNT/A in a medium containing 56 mM KCl and 2.2 mM CaCl2 to depolarize the neurons. Surprisingly, this also resulted in only a mild decrease (2–3 fold) of BoNT/A-induced SNAP-25 cleavage in both iCell Neurons and MSC cells, indicating that activity-dependent uptake of BoNT/A was only mildly inhibited by pre-exposure of the neurons to another BoNT serotype (Fig 2B). This is in agreement with a previous report, in which FM1-43 dye was taken up into BoNT/A intoxicated primary mouse spinal cord cells with chemical depolarization [19]. However, exposing the BoNT/A intoxicated cells to 5 rounds of depolarization prior to the dye uptake assay resulted in a failure to take up the dye, suggesting that the membrane available for endocytic uptake into BoNT/A intoxicated neurons is limited [19]. In contrast, subjecting BoNT/B exposed iCell neurons in which all VAMP2 was cleaved to 5 rounds of depolarization prior to BoNT/A exposure for 5 min with chemical depolarization of the cells resulted in only an about 3–4 fold decrease in BoNT/A activity compared to non pre-exposed cells. In addition, exposure of the same cells to BoNT/A for 24 h in the absence of depolarization resulted in a similar activity in pre-exposed and non pre-exposed cells (Fig 2C). This indicates that even after repeated depolarization, BoNT intoxicated neurons are capable of taking up further BoNT during fast depolarization dependent endocytosis, although at a 3-5-fold reduced rate, while BoNT/A uptake by slower, depolarization independent endocytosis is not altered. Taken together, these results suggest that BoNTs can be taken up independently of active synaptic vesicle exocytosis, although active synaptic vesicle recycling increases fast uptake of BoNTs. Screening of several selective endocytosis inhibitors further indicates that the uptake mechanism of BoNT/A into BoNT/B pre-exposed neurons as well as not pre-exposed neurons proceeds via clathrin dependent endocytosis (Fig 3), indicating that the endocytosis mechanism is not altered in BoNT intoxicated neurons.

Since the data revealed that several different BoNT serotypes can be taken up into the same neuronal cells, we next investigated whether exposure to two different BoNT serotypes may cause cytotoxicity to the cells. While only minimal and insignificant cell lysis was observed in cells intoxicated with BoNT/A, /B, /D, or /F as well as combinations of BoNT/A with BoNT/B, /D, or /F, there was significant cell lysis in cells exposed to BoNT/E (~10%) or combinations of BoNT/E and BoNT/B, /D, or /F (up to 30%) (Fig 4). This is in agreement with a recent report in which cytotoxicity was observed in rat hippocampal neurons exposed to BoNT/E [47]. It is noteworthy that in both studies the same preparation of BoNT/E was used, and it cannot be excluded that the cell lysis may be due to another co-purified factor present in the BoNT/E preparation. However, the data also indicated increased cell lysis in cells exposed to both, BoNT/E and BoNT/B, /D, and especially/F, which is in agreement with the hypothesis that cleavage of two SNARE proteins or a large portion of SNAP-25 may result in cell lysis [47]. This cell lysis could be caused by the lack of one or more functional SNARE proteins. However, it has been reported that primary mouse hippocampal neurons from both, VAMP 2 and SNAP-25 knockout mice, can be cultured and survive if seeded at a high enough density [48, 49], and even though mouse SNAP-25 or VAMP 2 mouse pups die upon birth, the embryos appear to develop normally until birth, except for some changes in appearance related to brown fat in VAMP 2 knock-out mice [49, 50]. Another possible explanation would be that truncated SNAREs or misfolded SNARE complexes may effect cell lysis, as has been suggested previously [51]. In either case, the observed cytotoxicity could be due to specific culturing conditions of the neurons such as a relatively low density or another as yet unknown toxin induced mechanism affecting 2 dimensionally cultured neurons. It is not known whether such cell lysis would also occur under different culture conditions or *in vivo*.

Exo- and endocytosis are considered to be tightly coupled in order to maintain the integrity of the cellular membrane [42, 52]. However, research in the past decade has suggested that endocytosis can proceed normally in the absence of exocytosis. Slow endocytosis has been shown to occur normally in VAMP 2 and SNARE knockout neurons [48, 53], whereas fast endocytosis is delayed in VAMP 2 knockout neurons [53]. In addition, it has been shown that compensatory endocytosis retrieves membrane that is different from that recently added by exocytosis [54–56]. It has previously been suggested that BoNT/A exposure of primary mouse spinal cord cells can result in an uncoupling of exocytosis and endocytosis, as endocytosis was not inhibited after BoNT/A treatment [19]. The data presented here indicate that this is also the case for BoNT/B, D, E, and F, and that uptake of BoNT into cultured neurons is independent of active synaptic vesicle recycling. Most botulinum neurotoxins utilize dual receptors for cell binding and entry consisting of gangliosides and the luminal domain of synaptic vesicle proteins [8]. The most likely explanation for display of the luminal domain of the syntaptic vesicle proteins on the cell surface is synaptic vesicle fusion. This occurs during active synaptic vesicle exocytosis, but also during spontaneous, action potential independent release, which takes place continuously [57]. Although SNARE proteins have been implicated in spontaneous vesicle release (reviewed in [57]), a recent report suggests that such release may occur by a mechanism distinct from action potential dependent release [58]. If some spontaneous synaptic vesicle release can take place independent of SNARE fusion, which would be blocked in BoNT pre-exposed neurons, subsequently added BoNTs could be taken up via receptor mediated endocytosis that utilizes synaptic vesicle membrane thereby exposed to the cell surface. Alternatively, synaptic vesicle proteins may be displayed on the cells surface by another not yet understood mechanism at a low level sufficient to support BoNT binding and endocytosis.

Finally, the quantitative uptake of BoNTs into neuronal cells previously intoxicated by BoNT suggests that atoxic BoNTs may serve as an ideal delivery vehicle for an intracellular botulism therapeutic that is directed at inhibiting the light chain. In addition, atoxic BoNTs may prove an attractive delivery vehicle for development of therapeutics towards other neuronal diseases in which exocytosis is inhibited.

## Supporting Information

S1 Fig Representative Western blots of SNARE cleavage in neuronal cultures pre-exposed to BoNTs. Human iPSC derived neurons (iCell Neurons) (a, b, c) or primary mouse spinal cord cells (d) were exposed to the following concentrations of BoNTs for 48 h: BoNT/A: 10 U / well / 50 μl, BoNT/B: 1000 U / well / 50 μl, BoNT/D: 1000 U / well / 50 μl, BoNT/E: 2000 U / well / 50 μl, BoNT/F: 30 U / well / 50 μl. After toxin removal, the pre-exposed or non pre-exposed cultures were then exposed to serial dilutions of BoNT/A (a, c, and d) or BoNT/B, /D, /E or /F (b) for 48 h. Cells were harvested by lysis in 1 x LDS lysis buffer and analyzed by Western blot. (a) Cleavage of VAMP2 as a result of BoNT/B, /D, and /F pre-exposure in hiPSC derived neurons, (b) Cleavage of SNAP-25 as a result of BoNT/A pre-exposure in hiPSC derived neurons. In image 3 cells were exposed to serial dilutions of BoNT/E as the second toxins, resulting in the appearance of the BoNT/E cleaved SNAP-25 band in both BoNT/A pre-treated and not pre-treated cells. (c) SNAP-25 cleavage as a result of BoNT/E pre-exposure in hiPSC derived neurons followed by exposure to serial dilutions of BoNT/A1. The non pre-treated cells show the BoNT/A cleavage pattern, whereas all SNAP-25 is converted to the BoNT/E cleaved form in the pre-exposed or simultaneously exposed cells. (d) Cleavage of VAMP-2 as a result of BoNT/B pre-exposure in MSC cells.
(PPTX)
