# Supplementary figures and images for: Botulinum Neurotoxins Can Enter Cultured Neurons Independent of Synaptic Vesicle Recycling
Source: PLoS One. 2015 Jul 24;10(7):e0133737. doi: 10.1371/journal.pone.0133737 (PMC4514655; doi:10.1371/journal.pone.0133737)

## Slide 1
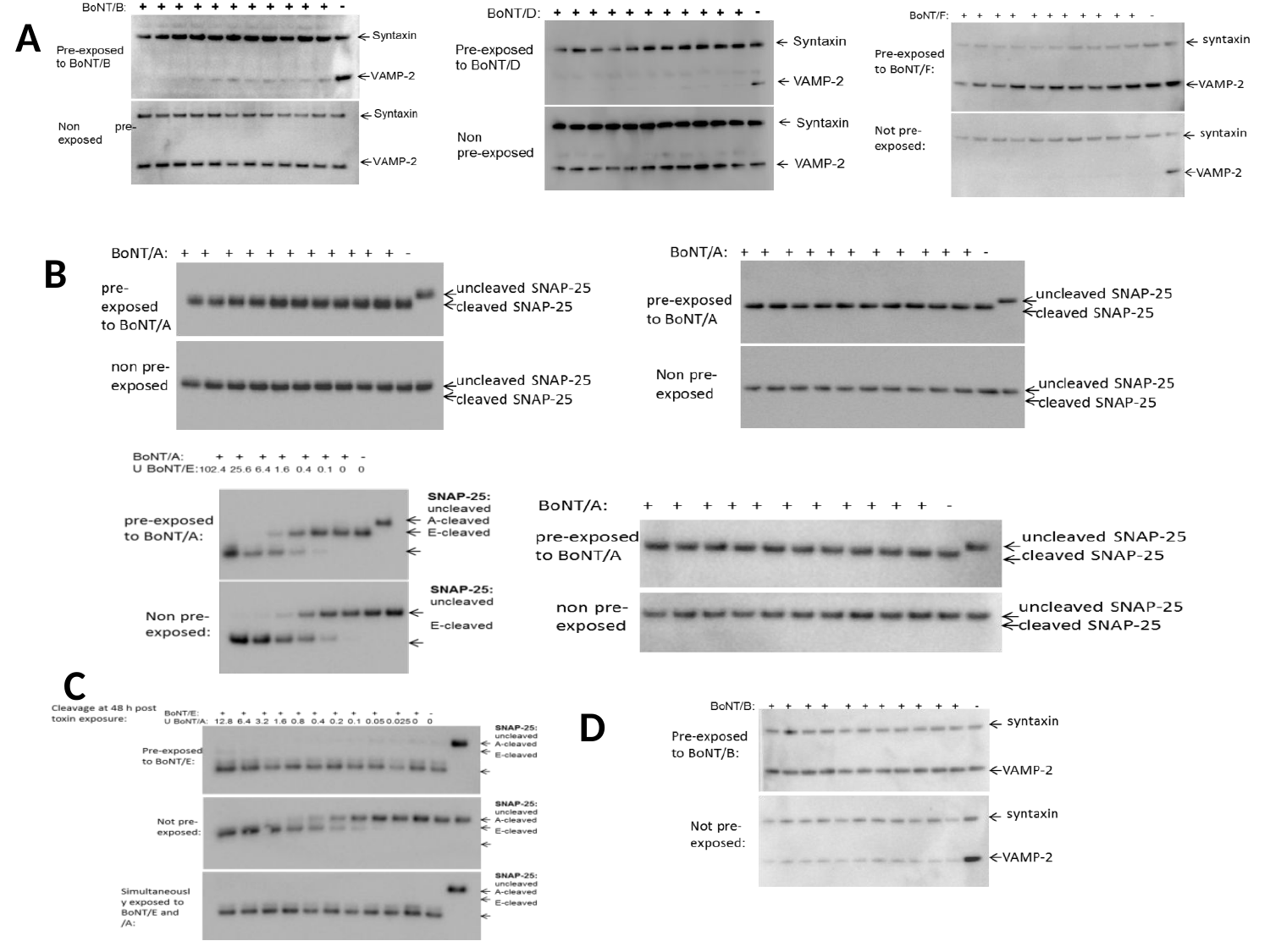

A
Supp Fig 1
B
C
D

Supplement: S1 Fig — Human iPSC derived neurons (iCell Neurons) (a, b, c) or primary mouse spinal cord cells (d) were exposed to the following concentrations of BoNTs for 48 h: BoNT/A: 10 U / well / 50 μl, BoNT/B: 1000 U / well / 50 μl, BoNT/D: 1000 U / well / 50 μl, BoNT/E: 2000 U / well / 50 μl, BoNT/F: 30 U / well / 50 μl. After toxin removal, the pre-exposed or non pre-exposed cultures were then exposed to serial dilutions of BoNT/A (a, c, and d) or BoNT/B, /D, /E or /F (b) for 48 h. Cells were harvested by lysis in 1 x LDS lysis buffer and analyzed by Western blot. (a) Cleavage of VAMP2 as a result of BoNT/B, /D, and /F pre-exposure in hiPSC derived neurons, (b) Cleavage of SNAP-25 as a result of BoNT/A pre-exposure in hiPSC derived neurons. In image 3 cells were exposed to serial dilutions of BoNT/E as the second toxins, resulting in the appearance of the BoNT/E cleaved SNAP-25 band in both BoNT/A pre-treated and not pre-treated cells. (c) SNAP-25 cleavage as a result of BoNT/E pre-exposure in hiPSC derived neurons followed by exposure to serial dilutions of BoNT/A1. The non pre-treated cells show the BoNT/A cleavage pattern, whereas all SNAP-25 is converted to the BoNT/E cleaved form in the pre-exposed or simultaneously exposed cells. (d) Cleavage of VAMP-2 as a result of BoNT/B pre-exposure in MSC cells. (PPTX) [file pone.0133737.s001.pptx]
